# Standardized porcine unilateral femoral nailing is associated with changes in PMN activation status, rather than aberrant systemic PMN prevalence

**DOI:** 10.1007/s00068-021-01703-2

**Published:** 2021-06-10

**Authors:** Michel Paul Johan Teuben, Roman Pfeifer, Klemens Horst, Tim-Philipp Simon, Marjolein Heeres, Yannik Kalbas, Taco Blokhuis, Frank Hildebrand, Leo Koenderman, Hans-Christoph Pape, Luke Leenen, TREAT-Research Collaboration, B. Auner, P. Störmann, B. Relja, I. Marzi, T. P. Simon, G. Marx, A. Haug, L. Egerer, M. V. Griensven, M. Kalbitz, M. Huber-Lang, R. Tolba, K. Reiss, S. Uhlig, K. Horst, M. Teuben, R. Pfeifer, K. Almahmoud, Y. Kalbas, H. Lüken, K. Almahmoud, F. Hildebrand, H. C. Pape

**Affiliations:** 1 Department of Traumatology, University Medical Center Utrecht, Heidelberglaan 100, 3584 CX Utrecht, The Netherlands; 2 Department of Traumatology, University Hospital Zurich, Raemistrasse 100, 8091 Zurich, Switzerland; 3 Laboratory for Translational Immunology, Heidelberglaan 100, 3584 CX Utrecht, The Netherlands; 4 Harald Tscherne Laboratory for Orthopaedic and Trauma Research, Raemistrasse 100, 8091 Zurich, Switzerland; 5 Department of Trauma and Reconstructive Surgery, RWTH University Hospital Aachen, Pauwelsstrasse 30, 52074 Aachen, Germany; 6 Department of Intensive Care and Intermediate Care, RWTH University Hospital Aachen, Pauwelsstrasse 30, 52074 Aachen, Germany; 7 Department of Surgery, University Hospital Maastricht, P. Debyelaan 25, 6229 HX Maastricht, The Netherlands; 8 Department of Pulmonology, University Medical Center Utrecht, Heidelberglaan 100, 3584 CX Utrecht, The Netherlands

**Keywords:** Intra-medullary nailing, Inflammation, Activation status, Polymorphonuclear neutrophils, Porcine modelling

## Abstract

**Purpose:**

Intramedullary nailing (IMN) of fractures is associated with increased rates of inflammatory complications. The pathological mechanism underlying this phenomenon is unclear. However, polymorphonuclear granulocytes (PMNs) seem to play an important role. We hypothesized that a femur fracture and standardized IMN in pigs is associated with altered appearance of PMNs in circulation and enhanced activation status of these cells.

**Methods:**

A porcine model including a femur fracture and IMN was utilized. Animals were randomized for control [anesthesia + mechanical ventilation only (A/MV)] and intervention [A/MV and unilateral femur fracture (FF) + IMN] conditions. PMN numbers and responsiveness, integrin (CD11b), L-selectin (CD62L) and Fc*γ*-receptor (CD16 and CD32)-expression levels were measured by flowcytometry of blood samples. Animals were observed for 72 h.

**Results:**

Circulatory PMN numbers did not differ between groups. Early PMN-responsiveness was retained after insult. PMN-CD11b expression increased significantly upon insult and peaked after 24 h, whereas CD11b in control animals remained unaltered (*P* = 0.016). PMN-CD16 expression levels in the FF + IMN-group rose gradually over time and were significantly higher compared with control animals, after 48 h (*P* = 0.016) and 72 h (*P* = 0.032). PMN-CD62L and CD32 expression did not differ significantly between conditions.

**Conclusion:**

This study reveals that a femur fracture and subsequent IMN in a controlled setting in pigs is associated with enhanced activation status of circulatory PMNs, preserved PMN-responsiveness and unaltered circulatory PMN-presence. Indicating that monotrauma plus IMN is a specific and substantial stimulus for the cellular immune system. Early alterations of circulatory PMN receptor expression dynamics may be predictive for the intensity of the post traumatic response.

**Supplementary Information:**

The online version contains supplementary material available at 10.1007/s00068-021-01703-2.

## Introduction

Trauma patients treated by intramedullary nailing (IMN) for a femoral shaft fracture are at increased risk to develop inflammatory complications such as in severe cases acute respiratory distress syndrome (ARDS) [1]. Despite improvements in trauma care, no changes in ARDS incidences in trauma cases occurred over the last three decades [2]. Nevertheless, a large number of studies indicate an association between intramedullary nailing of fractures and the occurrence of ARDS. Albeit underlying pathomechanisms are still poorly understood [3]. Previous trauma studies mainly determined the effect of humoral immune processes on ARDS development [1–6]. Plasma interleukin-6 (IL-6) levels are considered as a reliable marker for injury severity and it has been shown that femoral shaft fractures are associated with increased plasma IL-6 levels [6]. However, despite being investigated thoroughly, data on interleukins lack prognostic value for trauma conditions [7, 8]. To predict outcome and to gain insight in the pathomechanisms of orthopedic trauma related inflammatory complication, it is key to also investigate cellular immunological parameters. Cytokines, including interleukin-6, are involved in regulation of essential innate effector cells, namely polymorphonuclear leukocytes (PMNs) [7]. Previously, it has been demonstrated that post-trauma inflammation leads to mobilization and activation of circulatory PMNs. Activated circulatory PMNs migrate into the tissue compartment, including highly vascularized lung-tissue. Upon activation, tissue residing PMNs release radical oxygen species (ROS) and proteases, which can cause collateral damage to parenchymal cells and thereby contribute to organ dysfunction [4, 7]. Due to standardization issues in clinical trauma studies, the impact of IMN of long-bone fractures on PMN homeostasis remains unclear. We aimed to evaluate the impact of IMN of a femur fracture on appearance and activation status of the circulatory PMN population, in a standardized long-term large animal experiment, as it has previously been demonstrated that experimental IMN of a unilateral femur fracture is associated with enhanced pulmonary PMN pooling [9]. A porcine model was utilized due to the close similarity of anatomical, physiological, genetical and immunological properties to human [10]. We hypothesized that standardized IMN of a unilateral femur fracture in pigs is associated with increased appearance of PMNs in circulation and enhanced activation status of these cells.

## Material and methods

Experiments were executed in accordance with the German legislation governing animal studies following The Principles of Laboratory Animal Care [11]. The study was approved by the regional authority: Landesamt für Natur-, Umwelt- und Verbraucherschutz, LANUV NRW, Germany under application number: AZ 84-02.04.2014.A265. For these experiments, male *German landrace* pigs were included with a body weight between at 30 ± 5 kg. This study presents partial results obtained from a large animal porcine multiple trauma model. The model has been previously described in detail by Horst et al. [12].

### Study groups

For the purpose of the study two groups were composed:

i. *Control group*: anesthesia/mechanical ventilation only (A/MV), *n* = 6.
ii. *Intervention group* (FF + IMN): A/MV + femur fracture (FF) and intramedullary nailing, *n* = 6.

### Fracture model and operative intervention

Intramuscular premedication via injection of 4 mg/kg azaperone (Stresnil™, Janssen, Germany) was applied to animals prior to the experiments. Animals were pre-oxygenized with 100% O_2_/min. Anesthesia was maintained via continuous infusion of Propofol and Sufentanil. After intubation animals received volume controlled ventilation with lung-protective ventilation parameters (6–8 ml/kg/BW); i.e., inspiratory oxygen fraction (FiO_2_) of 0.5; positive end-expiratory pressure (PEEP) 8 mmHg (plateau pressure < 28 mmHg) and targeting a pCO_2_ of 35–45 mmHg (Draeger, Evita, Lübeck, Germany) as recommended for the chest trauma [13]. Ventilation was optimized based on continuous capnometry and frequent blood gas analyses.

An arterial line (Vygon, Aachen, Germany) was introduced in the femoral artery for continuous blood pressure assessment and a central venous catheter (Four-Lumen Catheter, 8.5 Fr., Arrow Catheter, Teleflex Medical, Germany) was placed in the external jugular vein for administration of fluids, anesthesia. The animals received a suprapubic urine catheter and fluid management could therefore be monitored on urine production measurements. The animal’s temperature was measured continuously and was kept between 38.7 and 39.8 °C, according to a physiologic temperature for this age group. Volume status was maintained by the infusion of Sterofundin^®^. Animals received antibiotics on a daily basis (Ceftriaxon^®^ 2 g, i.v.). All vitals were documented routinely during the observation period.

Control animals were instrumented and also kept under these conditions throughout the whole experiment but did not receive any trauma or surgery.

In the intervention group, a unilateral femur fracture was set using a bolt gun machine (Blitz-Kerner, turbocut JOBB GmbH, Germany) of which the bolt hit a custom-made plate positioned on the mid third of the femur. To produce a reproducible and standardized fracture, prior to fracturing a 5-cm skin incision was made and a channel towards the femur was created. Fracture placement was confirmed by X-ray imaging.

To simulate the rescue time, animals were observed for 90 min after fracturing the femur. During that time, volume management was reduced, O_2_ fraction was set to 0.21 to simulate ambient air and warming of the animals was suspended.

Thereafter, resuscitation was started in accordance with established trauma guidelines [14]. After a resuscitation time of 60 min, the operative phase started, and fracture fixation was performed by the placement of an intramedullary nail. All procedures were accomplished by one experienced orthopedic surgeon and under sterile conditions. Before nail placement, intramedullary reaming was performed, and adequate nail placement and fracture reduction were again confirmed by control X-ray imaging.

### Sampling

For blood PMN analysis, we collected peripheral blood from a central venous at several time points. The first sample (baseline) was drawn directly after the induction of general anesthesia. Thereafter we draw blood 6 h after intramedullary nailing and after 24, 48, and 72 h of observation.

### Peripheral blood PMN isolation and flowcytometry analysis

Analysis was performed with whole blood samples collected in a Vacutainer^®^ which was anticoagulated with Ethylenediaminetetraacetic acid (EDTA). An icecold isotonic NH_4_Cl-lysis buffer was utilized to lyse erythrocytes. Thereafter cells were washed with FACS-buffer (Dulbecco phosphate-buffered saline supplemented with 2% heat inactivated fetal calf serum, 5 mM EDTA). Antibody mixes were added, and samples had to incubate in a dark room for 45 min on 4 °C. After two wash steps with FACS, buffer cells were fixed by BD Cellfix (BD, Mountain View, CA, USA. All samples were analyzed within 8 h after collection using a FACS Canto II flowcytometer (BD, Mountain View, CA, USA). Data from individual experiments are depicted as fluorescence intensity as the mean fluorescence intensity (MFI) of at least 20,000 PMNs. PMNs were identified by specific forward- and sideward-scatter characteristics on CD45^+^-cells after exclusion of doublets (Supplement 1). Additionally, FL-8 (unstained Pacific Blue/autofluorescent)-positive cells were excluded. Sample processing and gating strategy have been validated before [15] and was further confirmed by pilot experiments including cell sorting experiments and subtype-specific co-expression analysis (including Swine Workshop Cluster (SWC)-8^+^/CD16).

### Determination of PMN-responsiveness to ex vivo LPS-stimulation prior and after trauma

In a different set of experiments (*N* = 8) from our research group (TREAT-consortium/ethical approval: ZH138/2017), early PMN receptor expression alterations upon ex vivo whole blood stimulation with the bacterial component lipopolysaccharide (LPS) was determined. A similar trauma model was utilized in these experiments as the initial model and the protocol is described in detail elsewhere [16]. For the ex vivo experiments, samples of monotrauma conditions (unilateral femur fracture + IMN) were obtained at baseline (BL) and 6 h after insult. Blood was collected using pyrogen-free heparinized Vacutainer^®^-tubes. Thereafter, whole blood was incubated with 10ug/ml (LPS, Sigma, Escherichia coli O55:B5) for 60 min at 37 °C. Unstimulated samples were analyzed to verify the effect of the LPS-stimulation. After processing of samples (as previously described), the single cell solutions were analyzed by flow cytometry. *PMN-responsiveness* was defined as the difference in PMN-CD11b expression between unstimulated and stimulated conditions at different timepoints. *Peak-PMN activation status* was defined as the level of PMN-CD11b expression upon ex vivo LPS-stimulation at different timepoints.

### Monoclonal antibodies utilized to determine activation status of blood PMNs

For the analysis of PMN receptor expression by flow measurements the following anti-pig antibodies were commercially purchased: CD45 (clone K252.1E4, Abd Serotec, Kidlington, UK), SWC8 (clone MIL-1, AbD Serotec, Kidlington, UK), CD11b(RIII) (clone 2F4/11, AbD Serotec, Kidlington, UK), CD16 (clone G7, Abd Serotec, Kidlington, UK), CD62L (clone produced by Laboratory for Translational Research, Utrecht) and CD32 (Clone AT10, AbD Serptec, Kidlington, UK).

### Absolute cell counts

CountBright counting beads (Invitrogen, Waltham, Massachusetts, USA) were utilized to calculate absolute cell numbers.

### Statistical analysis

Pooled data from individual experiments were described by mean and standard deviation (SD) or by median and interquartile range (IQR). For comparisons, a Students *T* test, Paired-Samples *T* tests or Mann Whitney *U* test were used as appropriate. Furthermore, changes over time were analyzed using repeated measurement analysis by the non-parametric hypothesis Wilcoxon signed-rank test. A *P* value < 0.05 is considered statistically significant. Data were analyzed using software programs SPSS version 17.0 (SPSS Inc., Chicago, IL, USA) and GraphPad Prism (GraphPad Software Inc., La Jolla, CA, USA).

## Results

All experimental animals survived the 72 h observation period. Furthermore, a mid-shaft fracture and adequate fracture fixation was confirmed by macroscopic analysis after termination in all intervention animals. No complications were diagnosed during the observation period.

### Absolute PMN-numbers in peripheral blood over time

**Fig. 1:**
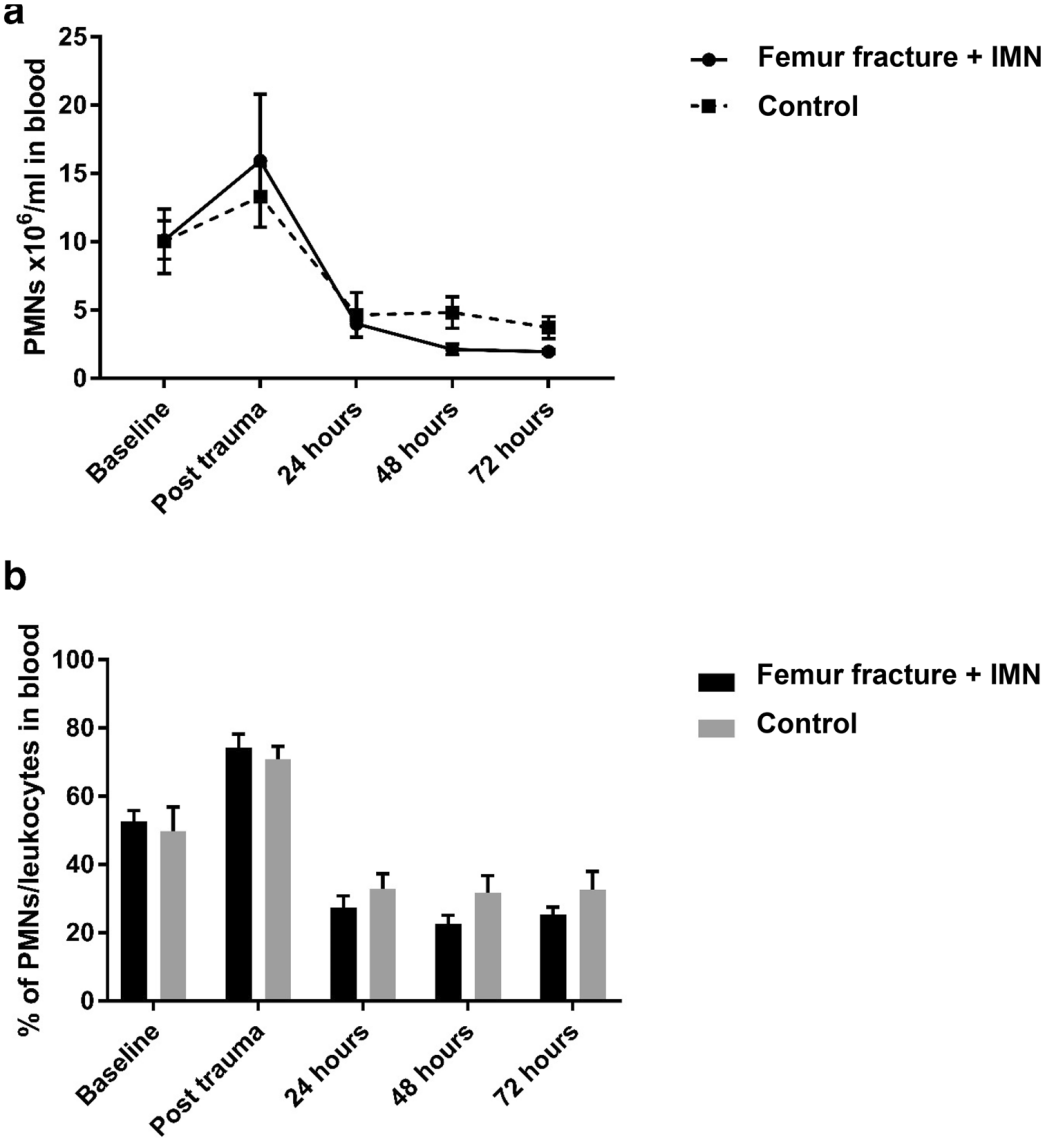
**a** Absolute neutrophil numbers in peripheral blood over time. **b** Alterations in percentages of PMNs/leukocytes in circulation

No statistically significant differences in circulatory PMN numbers were found between groups prior, and immediately after intervention [control 10.5 (IQR 4.8–15.0) × 10^6^ cells/ml vs. FF + IMN 10.3 (IQR 7.4–12.7) × 10^6^ cells/ml, *P* = 1.00]. In addition, no differences between the groups were present 6 h after the intervention-timepoint [control 13.6 (IQR: 8.4–18.1) × 10^6^ PMNs/ml and fracture + IMN 16.3 (IQR 6.4–25.1) × 10^6^ cells/ml, *P* = 0.73]. Thereafter a gradual and statistically significant decrease in circulatory PMN numbers was seen in both groups (*P* < 0.01). After 3 days of observation polymorphonuclear leukopenia was seen in both groups, however PMN counts did not differ between control [3.0 (IQR 2.1–5.7) × 10^6^ cells/ml] and FF + IMN-conditions [2.0 (IQR1.6–2.2) × 10^6^ cells/ml, *P* = 0.11]. Circulatory PMN numbers are shown in Fig. 1a.

As displayed in Fig. 1b, under homeostatic conditions. No statistical differences were seen between control and intervention-groups, with respectively 58.9 (IQR 33.0–62.1)% blood white blood cells (WBCs) were identified as PMNs and 57.3 (IQR 44.8–58.0)% PMNs/WBCs (*P* = 0.69). After intervention, the percentage of circulatory PMNs/all leukocytes peaked in both control (*P* = 0.03) and the FF + IMN (*P* = 0.008) groups. No differences between groups were seen at this timepoint. Thereafter, the percentage of circulatory PMNs dropped in both study groups. At 2 days of observation, lowest circulatory PMN percentages were encountered and again, percentages between control [24.0 (IQR 23.2–44.0)%] and FF + IMN [25.0 (IQR 17.6–25.9)%] conditions did not differ significantly.

### Membrane receptor expression of Mac-1 (CD11b) and L-selectin (CD62L) on circulatory PMNs over time

**Fig. 2:**
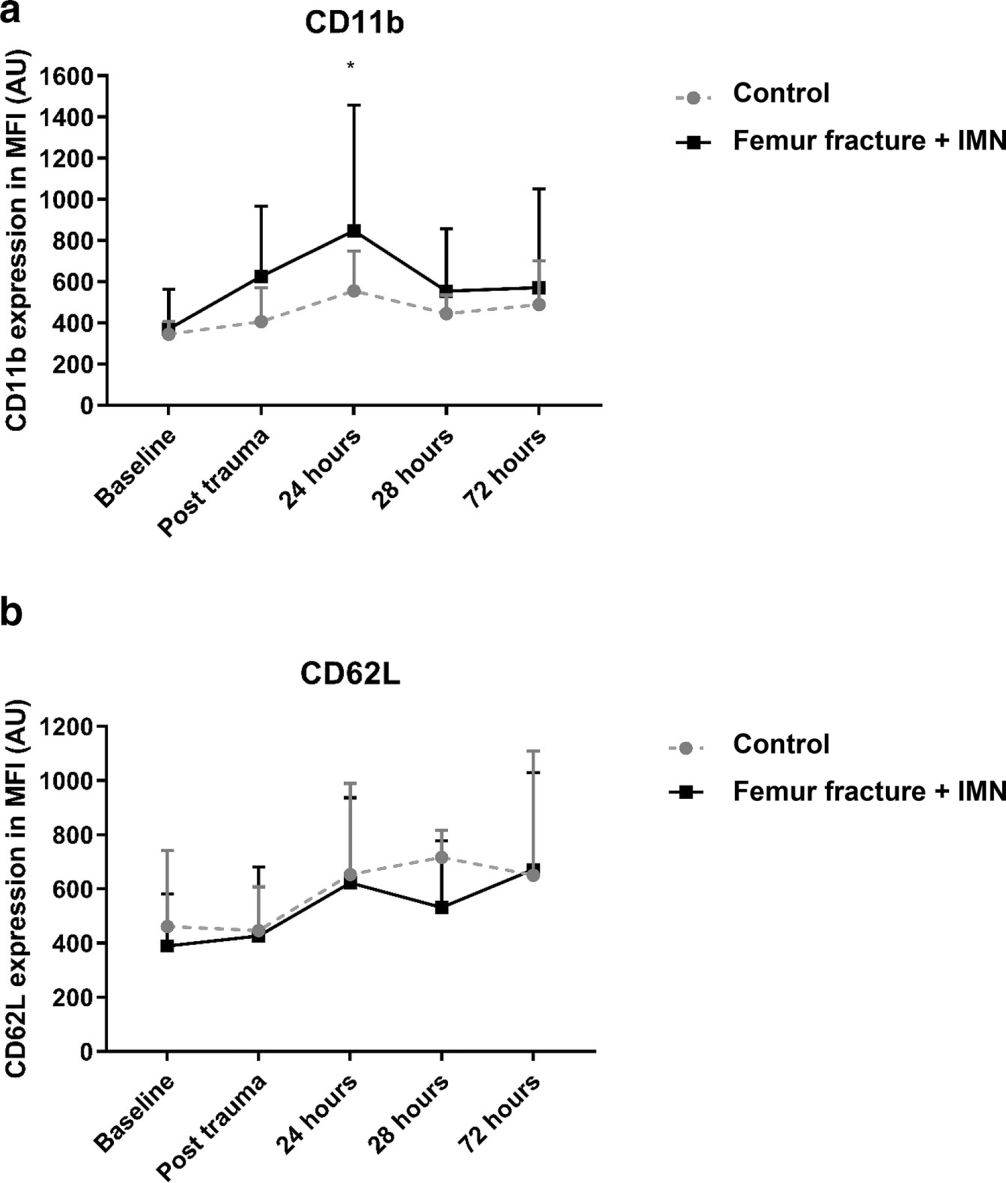
**a** Circulatory neutrophil Mac-1 expression changes over time. Receptor expression on neutrophils over time. *P < 0.05. **b** Circulatory neutrophil L-Selectin expression alterations over time. Receptor expression on neutrophils over time. Data in mean (SD). *P < 0.05, control vs. isolated femur fracture and IMN

Figure 2a displays alterations in membrane receptor expression of CD11b on circulatory PMNs over time. Significantly increased blood PMN surface expression of CD11b was found in animals exposed to FF + IMN compared with control conditions. Upon intramedullary femoral nailing, expression levels of CD11b on blood PMNs increased significantly during the first day and peaked at 24 h of observation. Thereafter, PMN-CD11b decreased gradually over time and returned to baseline values within 3 days after insult. In contrast, cell surface CD11b expression on circulatory PMNs in control animals did not change significantly over time.

After 1 day of observation, CD11b expression levels on blood PMNs were twice as high in intervention animals as in control conditions (*P* = 0.016).

A non-significant trend of gradual increasing L-selectin cell surface expression on circulatory PMNs over time occurred in both control and IMN groups, however no statistical differences between groups were found at any timepoint (Fig. 2b).

### Membrane receptor expression of Fcγ-receptors on blood PMNs.

**Fig. 3:**
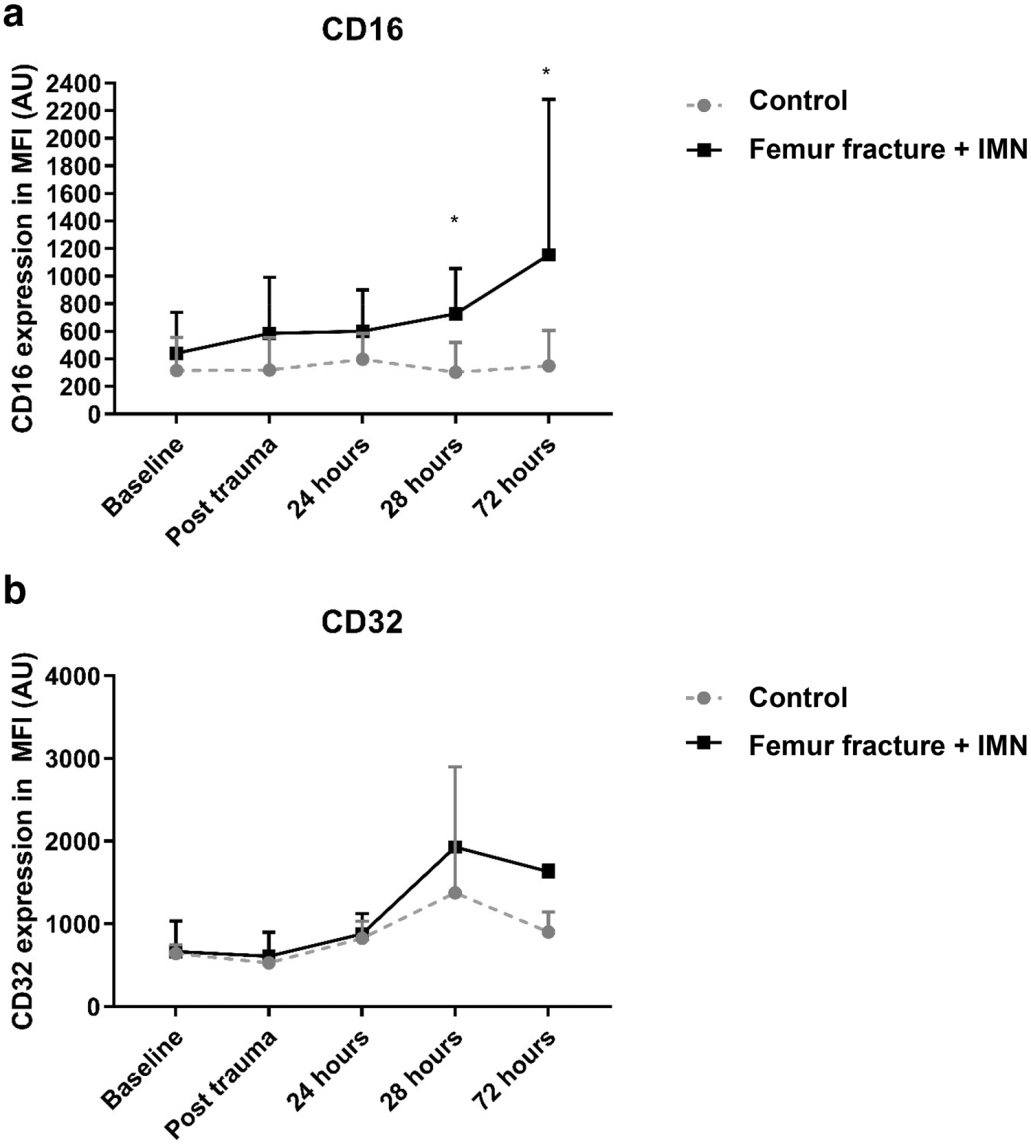
**a** Circulatory neutrophil Fc*y*RIII expression alterations over time. **b** Circulatory neutrophil Fc*y*RII expression alterations over time. Receptor expression on neutrophils over time. Data in mean (SD). *P < 0.05, isolated femur fracture and IMN vs. control

Blood PMN-CD16 (Fc*γ*III) expression significantly increased 2 and 3 days after trauma (FF + IMN-group) (*P* = 0.016 and *P* = 0.032, respectively), whereas PMN-CD16 expression in the control group was found to be unaltered over time. During the course of the experiment, cell surface expression levels of CD32 (Fc*γ*II) on circulatory PMNs did not differ significantly between control and intervention groups. Fc*γ-*receptor changes over time are shown in Fig. 3.

### PMN-responsiveness to ex vivo lipopolysaccharide stimulation at baseline and after insult

**Fig. 4:**
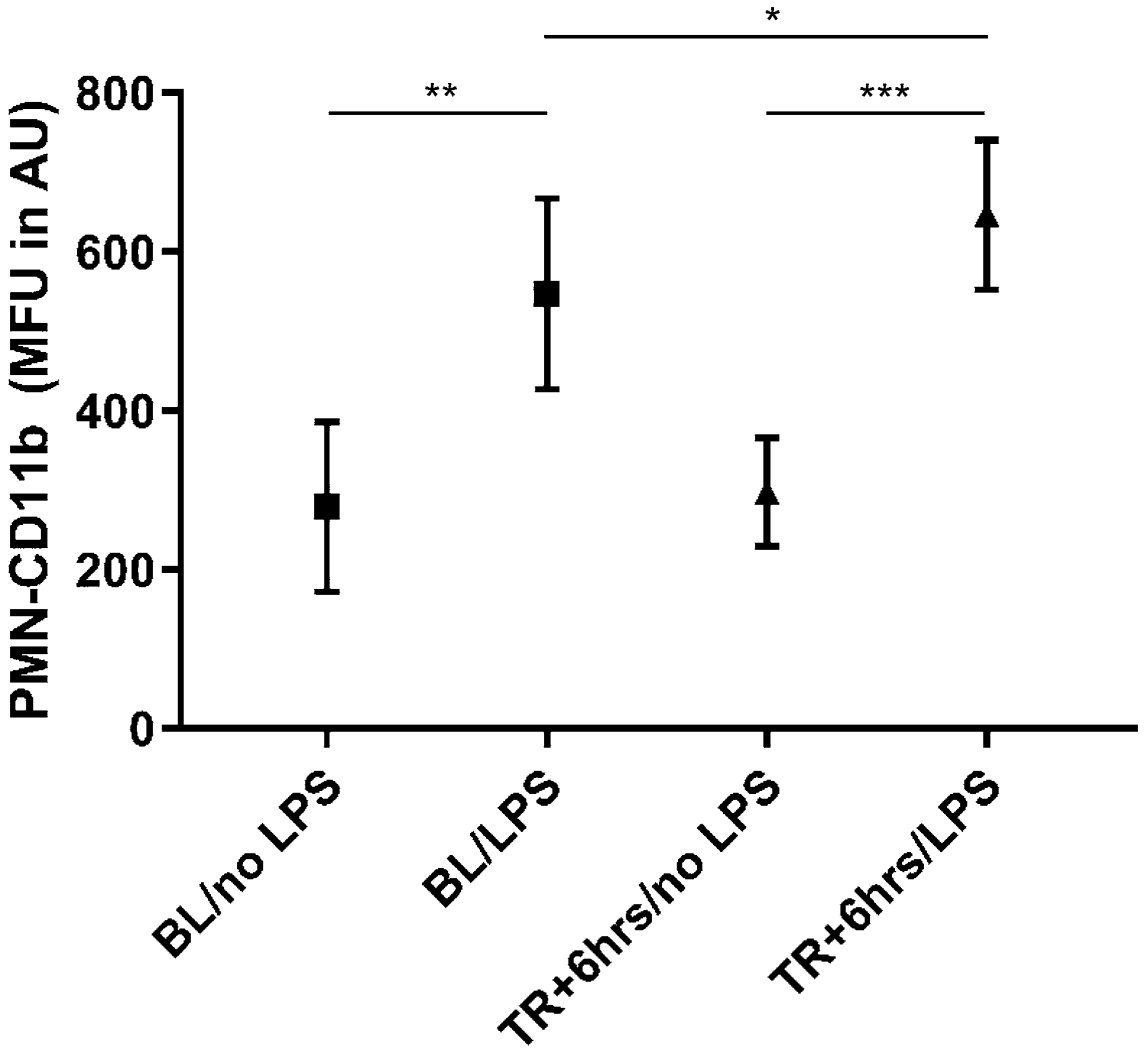
PMN-Mac 1 expression following ex vivo LPS-stimulation prior and post instrumentation. Data in mean (SD). *P < 0.05, **P < 0.01, ***P < 0.001

No statistically significant differences were seen between unstimulated conditions. PMN-responsiveness was preserved over time as ex vivo LPS-stimulation evoked a statistically significant increase of PMN-CD11b expression after stimulation both at baseline (mean ± SD increase of CD11b-MFI 127.5 ± 107.6%, *P* = 0.002) and 6 h after insult (mean ± SD increase of CD11b-MFI 130.7 ± 71.1%, *P* = 0.0002). Moreover, peak-PMN activation status, reflected by the amplitude of PMN-CD11b expression after ex vivo LPS-stimulation was significantly higher after insult than at baseline (with a mean ± SD difference of CD11b-MFI 21.0 ± 19.2%, *P* = 0.03). Results of ex vivo LPS-stimulation of peripheral blood samples at baseline and after insult are displayed in Fig. 4.

## Discussion

Orthopedic trauma and intramedullary nailing are associated with increased incidences of inflammatory complications [1]. Polymorphonuclear leukocytes are believed to play an important role in the development of these complications after trauma. Clinical trauma studies, however, lack standardization due to (i) heterogeneous insult condition types and severity, (ii) suboptimal timing of sampling/procedures, (iii) variation in patient characteristics (genetic background, co-morbidities, physiology, age, gender, medication). Therefore, the current standardized large animal long-term observation study was executed, and the results may be summarized as follows:

1. General anesthesia and mechanical ventilation in pigs in a controlled context is associated with changes in circulatory PMN numbers and cell surface receptor dynamics over time.
2. Standardized experimental femur fracturing and subsequent intramedullary nailing evoke a more intensified systemic PMN response which is characterized by increased early activation status of circulatory PMNs, rather than altered circulatory PMN counts or early impaired PMN-responsiveness.

This indicates that a unilateral femur fracture and subsequent intramedullary femoral nailing should be considered as a substantial trigger for the innate cellular immune system.

In addition, we previously managed to demonstrate with this porcine monotrauma model that, compared with control conditions, IMN of an isolated femur fracture is associated with increased pulmonary PMN occurrence after 72 h [9]. This indicates that early alterations of circulatory PMN activation status (current study) precede pulmonary innate immune cell accumulation [9]. It is tempting to speculate that monitoring of circulatory PMN alterations could assist in the prediction of remote organ inflammation.

Post-insult variations in circulatory PMN numbers over time in our study mimic both clinical and experimental findings [17–19]. Furthermore, in line with a rodent trauma study, we found altered circulatory PMN presence over time in both the control (A/MV) and intervention study groups. This suggests that general anesthesia and mechanical ventilation shape circulatory PMN kinetics and that the additional impact of trauma/surgery on changes in circulatory PMN presence is limited [20]. Despite the presumed limited additional impact of a unilateral fracture and subsequent fracture fixation on circulatory PMN numbers, the current study does reveal that cell surface expression profiles of relevant receptors are largely affected by this trigger (FF + IMN). This makes it tempting to hypothesize that the interplay between (i) trauma anesthesia/mechanical ventilation settings and (ii) fracture (fixation standards) dictates the innate immune response in these trauma patients. All these factors and their interplay should therefore be considered as potential targets for immune modulation and this requires further study.

Nowadays, cell surface receptor expression, in contrast to immune cell functionality, can easily be determined by modern automatized flow systems [21]. Determination of these parameters in a controlled setting is of great relevance and may form the basis for future routine diagnostic, prognostic and therapeutic investigations in the clinical setting. Therefore the current study focusses on PMN activation status by studying well-established PMN markers with appropriately defined biological function [22], rather than ex vivo characterization of PMN functionality.

CD11b is a well-established activation marker and in vitro studies demonstrate that cell activation is associated with neutrophil-CD11b upregulation [23, 24]. Clinical studies on systemic inflammation further showed that circulatory neutrophil-CD11b expression rise upon insult [25–27]. Like the findings of the current study, Johansson et al. demonstrated increased PMN-CD11b expression in burn patients [25] and others demonstrated an early rise of neutrophil-CD11b cell surface expression in trauma patients [26]. In addition, a similar pattern of Mac-1 expression kinetics was seen by Botha et al. [27]. They showed that neutrophil-CD11b expression levels in trauma patients peaked between 6 and 18 h after admission [27]. In the current porcine experiment, PMN-CD11b expression reached peak levels later, namely after 24 h. We believe that this discrepancy may mainly be due to differences in trauma load between both studies. Trauma victims in the study from Botha et al. had ISS scores over 20 [27], whereas our porcine trauma model represents a monotrauma model with an ISS of 9. This might imply that trauma severity is associated with PMN-integrin expression.

A study from Bathia et al. further found that PMN-CD11b expression started to decrease and return to homeostatic levels, as late as 72 h after severe trauma [28]. A similar pattern of restoration was observed in our experimental study. Timing of definitive fracture fixation for orthopedic trauma is a topic of considerable debate. Operative intervention is an additional trigger for the immune system and thereby leads to an upsurge of the post-trauma inflammatory response [29]. As integrins are considered as essential receptors in PMN adhesion and migration processes, changes in circulatory PMN-CD11b expression may potentially be utilized to guide timing of definitive fracture fixation. Especially as homogeneous blood PMN-CD11b response was found in all previously mentioned trauma studies and our experimental data. This response entails peak PMN-Mac-1 expression levels within the first 24 h and restoration to homeostatic levels within 72 h post-insult. Therefore, it is tempting to speculate that postponing IMN for the first 72 h in the critically ill is beneficial as integrin levels are allowed to normalize first, which is in line with clinical studies.

L-selectin plays an critical role in neutrophil transmigration as this receptor binds to its ligands on vascular endothelium and thereby facilitates slowing down, or ‘rolling’ of neutrophils [30]. Neutrophil activation is typically characterized by shedding of L-selectin [31] and higher trauma load is associated with more profound decreased neutrophil L-selectin expression [32]. In the current study, L-selectin expression on PMNs did not significantly change over time after insult. A similar observation was made in a study by Mommson et al. on patients undergoing elective surgery of the lower extremities [33] and in a cohort of burn patients [25]. Striking alterations in blood neutrophil L-selectin expression over time, however, have been shown in a study performed by Maekawa et al. on more severely injured trauma patients [34]. These discrepancies might be partly explained by differences in trauma load and the intensity of the post-insult inflammatory response and are thereby in line with the observations from Seekamp et al. [32].

Fc*γ*-receptors are involved in activation of neutrophils as they bind to immunoglobins (IgG) either in aggregates or attached to pathogens. Binding induces phagocytosis or promotes oxidative burst [35]. Interestingly, expression of Fc*γ*III (CD16) on blood PMNs increased over time in our model. This contradicts kinetics in burn injuries [25, 36] and polytrauma patients studied by White-Owen et al. in which overall PMN-CD16 expression dropped after trauma and remained decreased for more than 48 h [37]. We believe that our porcine monotrauma insult-condition did not cause an immune response that was intense enough to evoke the mobilization of novel subsets, and more specifically CD16^low^- PMNs in mobilization [38, 39]. Consequently, altered blood PMN-pool constitution-related reduction of PMN-CD16 expression did not occur in our model. The paradoxical increase of PMN-CD16 expression in our trauma study might reflect a specific activation profile of the blood neutrophil pool as regulation of the PMN-CD16-receptor in trauma is affected by different processes including altered PMN-phenotype appearance, delayed apoptosis, potentially modified balance between receptor shedding/biosynthesis and mobilization of the PMNs´ internal pool of pre-formed CD16 [38–41]. However most likely an interplay between these mechanisms occurs and, in our view, PMN-CD16 receptor dynamics upon trauma should be a focus of upcoming studies.

Our findings regarding PMN-Fc*γ*II (CD32) expression changes are in accordance with a study from Visser et al. Both studies showed no evident alterations in PMN-CD32 expression within the first 24 h after trauma [42].

The current study demonstrates that a more profound blood PMN-CD11b/CD16-response was found in the group subjected to orthopedic trauma/IMN than in animals exposed to anesthesia and mechanical ventilation only. Thereby our experimental study reveals that orthopedic trauma and subsequent IMN elicit a specific pattern of enhanced PMN-CD11b/Mac-1 cell surface expression. This may be an important factor in the association between increased occurrence of inflammatory complications and intramedullary nailing in orthopedic trauma. It is tempting to speculate that IMN for long-bone fractures induces a specific systemic PMN activation profile due to increased release of DAMPs, local cytokines, pro-inflammatory bone particles or angiogenic/growth factors [5–7, 43, 44]. Upcoming experimental studies should focus on the identification of involved pathways.

In contrast to studies on polytrauma [45, 46] we did not observe impaired PMN-responsiveness as LPS-induced upregulation of CD11b on PMNs was retained after controlled monotrauma and fracture fixation. Interestingly, there even seems to be a trend towards intensified early PMN-responsiveness upon trauma and subsequent nailing as peak-PMN activation status, reflected by the amplitude of PMN-CD11b expression after LPS-stimulation, is significantly greater after trauma than under homeostatic conditions. This phenomenon is in contrast with observations in polytrauma studies. As in these clinical investigations, impaired PMN-responsiveness, and the occurrence of refractory neutrophils in circulation are linked with severe trauma and complicated courses. [45–47].

The specific impact of instrumentation on early PMN-receptor alterations was studied as well. In short, additional samples were collected prior to fracture fixation and 2 h after fracture fixation. In line with Hietbrink et al. [26] no differences in circulatory PMN-integrin/selectin/Fc*γ*- receptor expression were seen between pre- and post-instrumentation conditions. (Data are displayed in Supplementary file 2).

The current standardized porcine trauma model, and human trauma studies are (except for CD16-data) remarkably similar regarding PMN receptor dynamics trends. Given these similarities, the current porcine trauma model is very suitable for proof-of-principle interventions with novel therapeutic strategies for trauma. Integral translational effects are summarized in Supplementary file 3.

Limitations of this study include its sample size, however relevant effects reached statistical significance and, therefore, we conclude that there was no need to include/sacrifice more experimental animals. We further decided not to include additional study conditions without clinical importance or translational value. So, unconventional fracture fixation techniques for cardiopulmonary compensated monotrauma scenarios (such as external fixation/plating/splinting) or IMN without fracture induction were not investigated. Finally, as porcine immune cells, in contrast to rodent models [48], do allow for adequate analysis of the key Fc*γ*-receptor family, we decided to focus with our antibody panel on these specific markers plus integrins and selectins, rather than adding antibodies aimed to investigate other circulatory immune cell populations.

In conclusion, the current standardized large-animal study demonstrates that general anesthesia and mechanical ventilation in pigs is associated with changes in circulatory PMN numbers and activation status. Furthermore, an isolated femur fracture and intramedullary nailing is associated with more intensified activation of circulatory PMNs and preserved PMN-responsiveness. Indicating that monotrauma plus IMN should be considered as a substantial stimulus for the cellular immune system with a specific profile of enhanced circulatory PMN-integrin/Fc*γ*RIII-receptor expression. This mechanism may play an important role in the development of inflammatory complications upon IMN for long-bone fractures and requires further research. Moreover, analysis of early circulatory PMN receptor expression profiles may be useful to predict the intensity of the long-term post-insult immune response and may thereby be an interesting tool to guide therapy decisions in trauma patients.

## Supplementary Information

Below is the link to the electronic supplementary material.

Supplementary file1 (DOCX 92 KB)

Supplementary file2 (DOCX 210 KB)

Supplementary file3 (DOCX 15 KB)

## Data Availability

The datasets generated and analyzed during the current study are not publicly available. However datasets are available from the corresponding author on reasonable request.
