# Supplementary material for: Standardized porcine unilateral femoral nailing is associated with changes in PMN activation status, rather than aberrant systemic PMN prevalence
Source: Eur J Trauma Emerg Surg. 2021 Jun 10;48(3):1601–11. doi: 10.1007/s00068-021-01703-2 (PMC9192391; doi:10.1007/s00068-021-01703-2)
Supplement: Supplementary file 1 — Supplementary file1 (DOCX 92 KB) [file 68_2021_1703_MOESM1_ESM.docx]

Supplement 1: **Flowcytometry: 3-step gating strategy for PMN identification**

**Step 1**: *PMN gating by FSC-SSC
signal characteristics on CD45+ cells*

**Step 2**: *Exclusion of doublets*


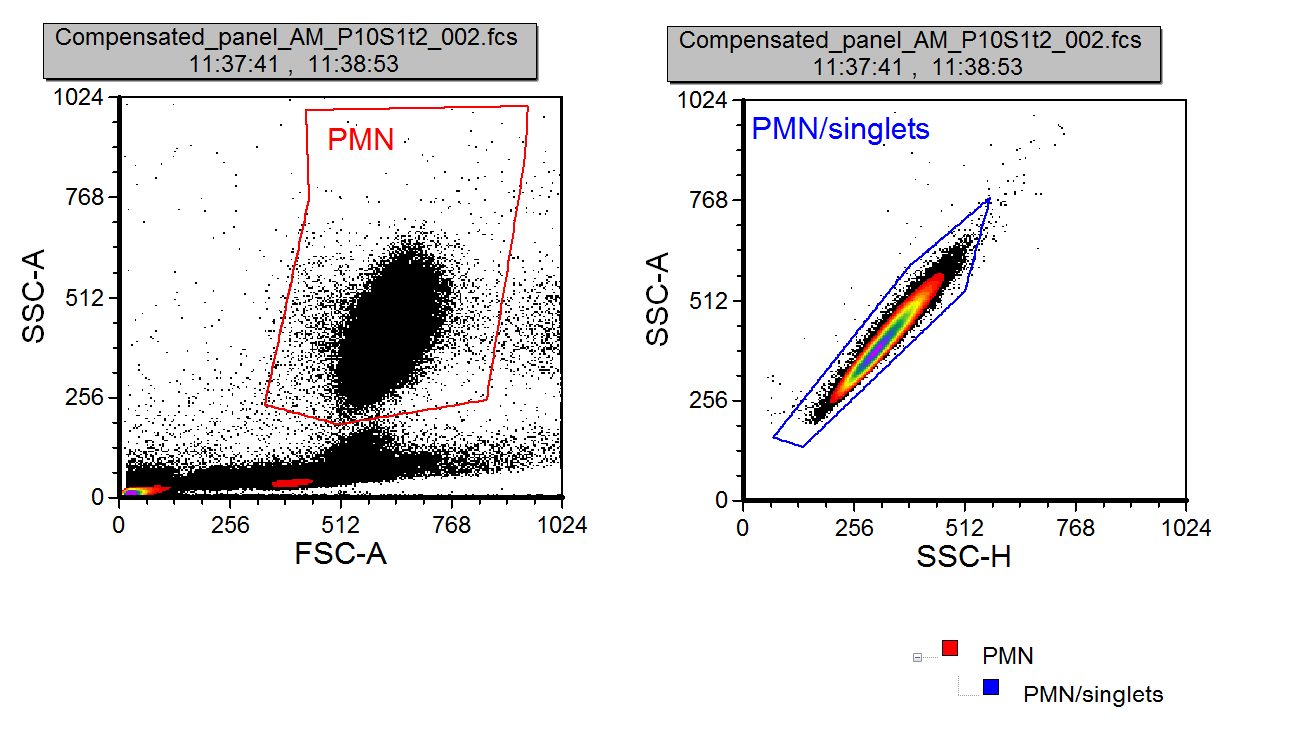


CD16 (FcyRIII)

**Step 3:***Validation by cell surface expression levels of CD16 and SWC8 on PMN-gate vs. Lymphocytes/Monocyte-gate*


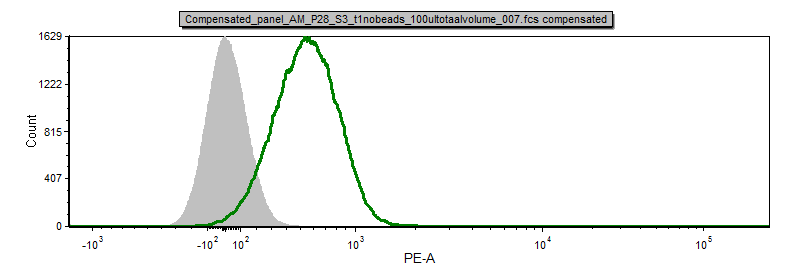
PMN-FcyRIII
(CD16) expression

PMN gate
Lymphocytes/
Monocytes gate

Swine
Workshop
Cluster-mAB-1

PMN gate
Lymphocytes/
Monocytes gate


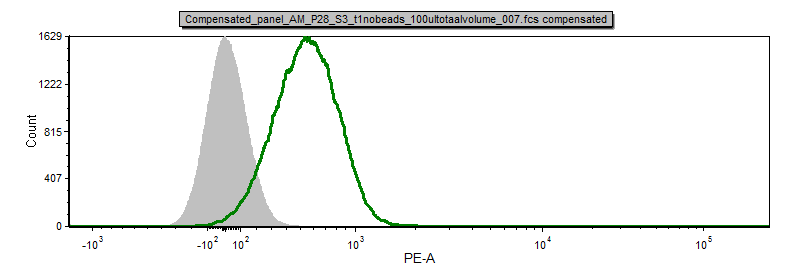
PMN-SWC-8
expression
