# Supplementary material for: Standardized porcine unilateral femoral nailing is associated with changes in PMN activation status, rather than aberrant systemic PMN prevalence
Source: Eur J Trauma Emerg Surg. 2021 Jun 10;48(3):1601–11. doi: 10.1007/s00068-021-01703-2 (PMC9192391; doi:10.1007/s00068-021-01703-2)
Supplement: Supplementary file 2 — Supplementary file2 (DOCX 210 KB) [file 68_2021_1703_MOESM2_ESM.docx]

Supplement 2: **PMN-cell surface receptor alterations pre- and post-instrumentation**

**
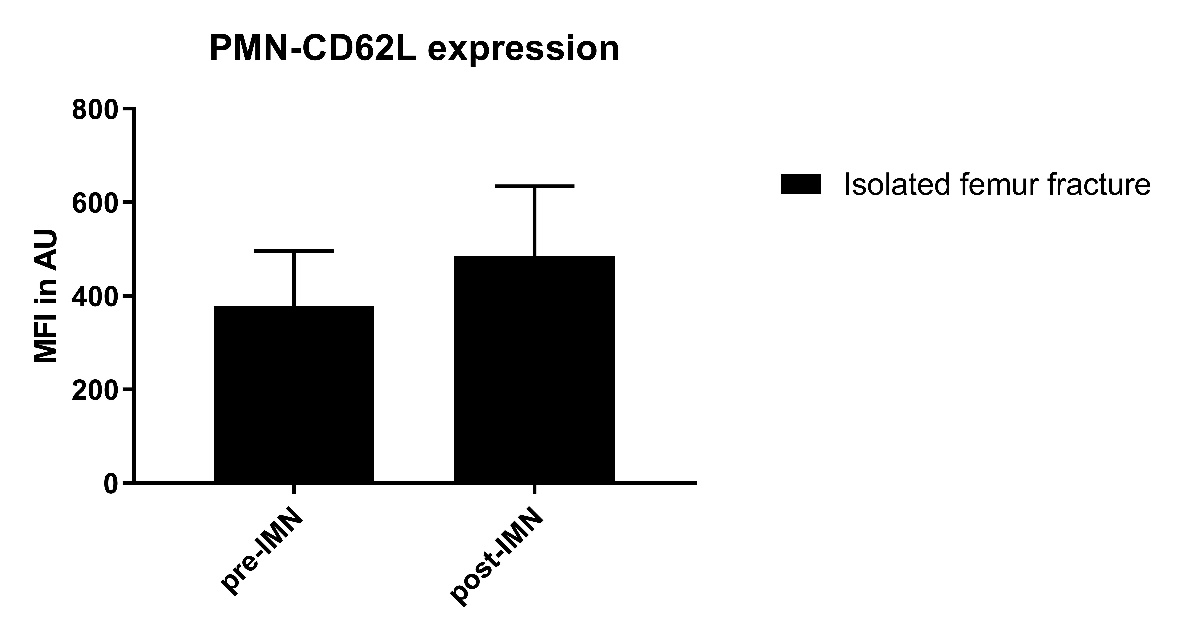

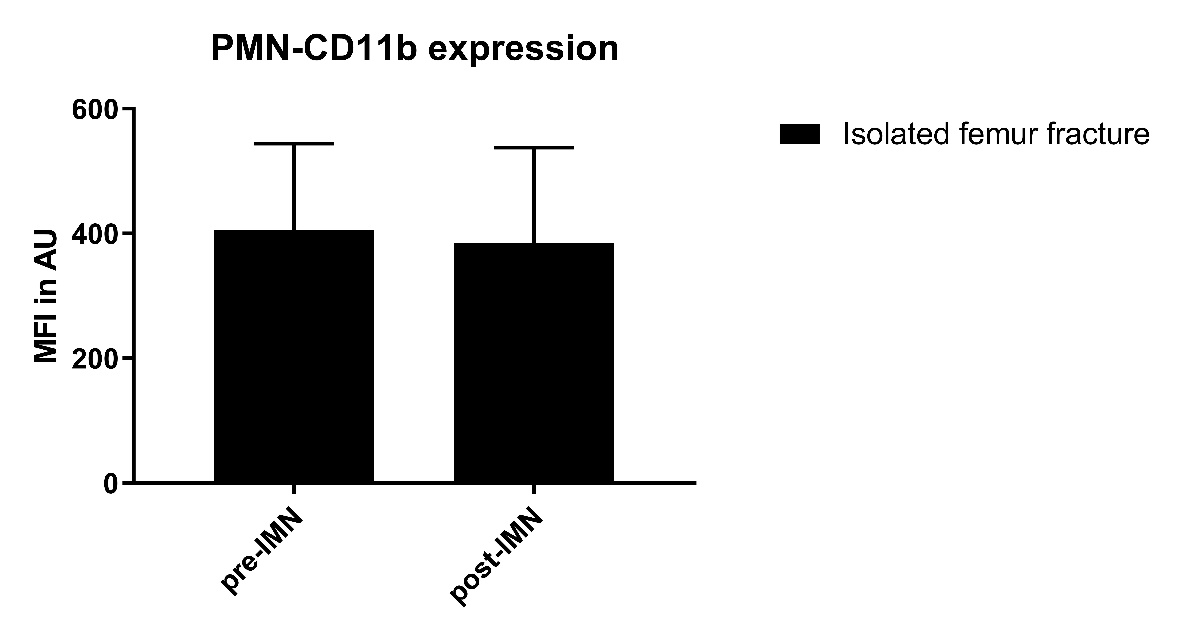
**

**
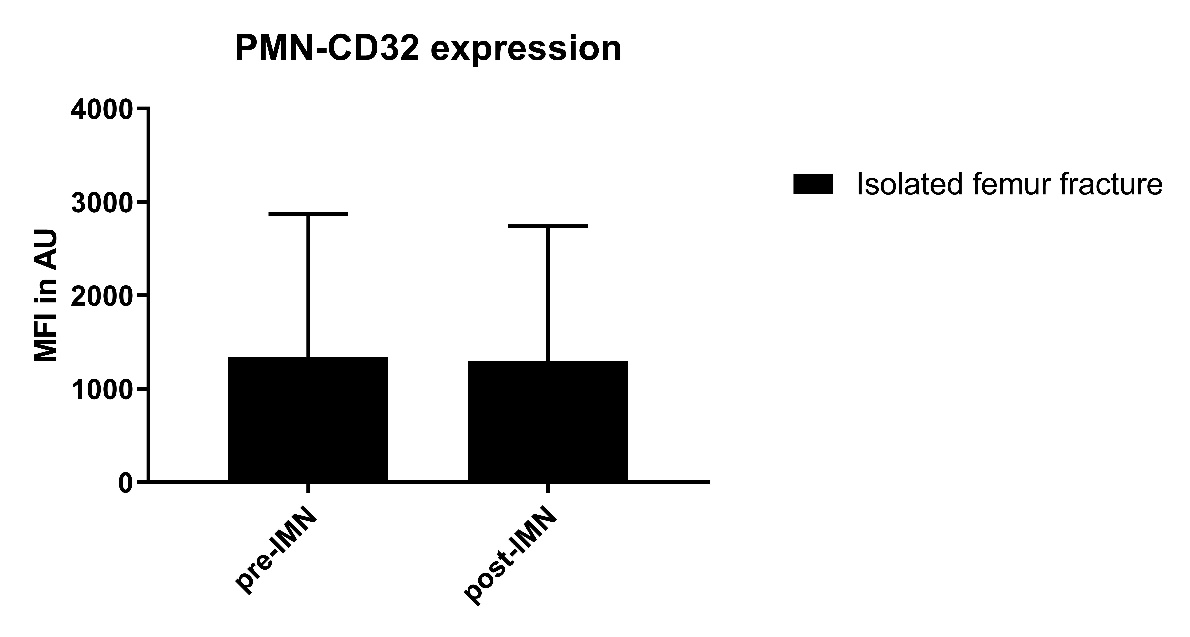

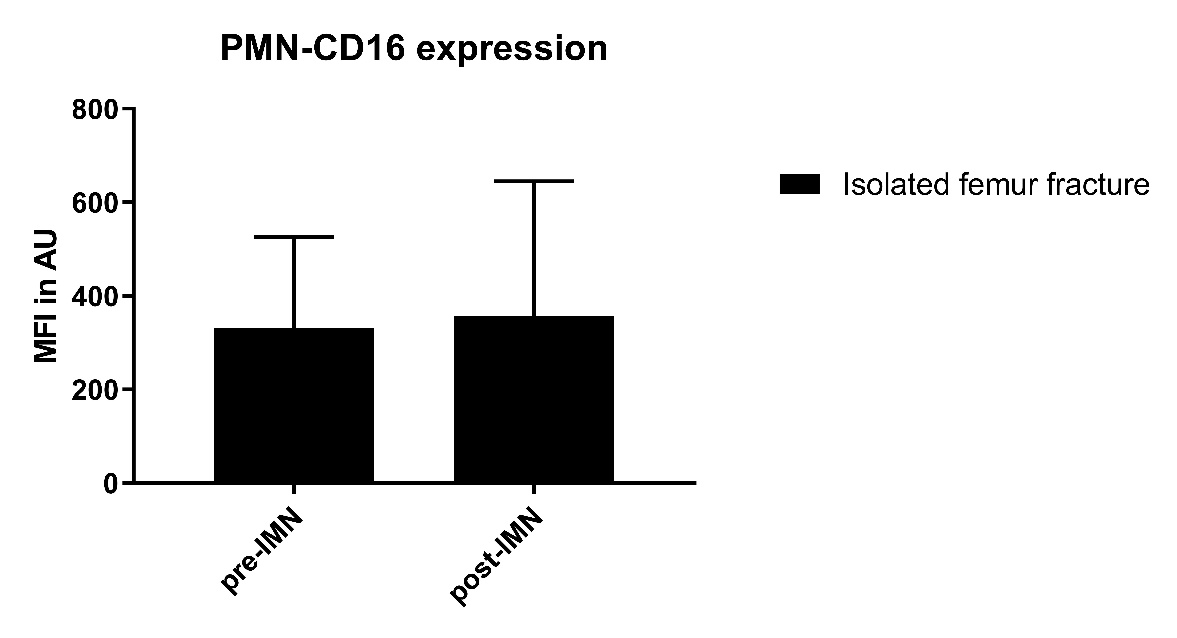
**

*Receptor expression on PMNs prior to operative intervention (pre-IMN) and 2hrs after operative intervention (post-IMN).* Data in mean (SD). *No significant differences were seen between conditions*
