# Supplementary material for: Standardized porcine unilateral femoral nailing is associated with changes in PMN activation status, rather than aberrant systemic PMN prevalence
Source: Eur J Trauma Emerg Surg. 2021 Jun 10;48(3):1601–11. doi: 10.1007/s00068-021-01703-2 (PMC9192391; doi:10.1007/s00068-021-01703-2)
Supplement: Supplementary file 3 — Supplementary file3 (DOCX 15 KB) [file 68_2021_1703_MOESM3_ESM.docx]

Table 1: **Overview of human and standardized porcine trends in neutrophil cell surface expression upon trauma**

| Cell surface receptor | Integral effect upon activation | Human cell surface receptor dynamics | Porcine cell surface receptor dynamics |
| --- | --- | --- | --- |
| CD11b (Mac-1) | **↗** | Early increase upon burns [25]/trauma [26,27] | Early rise upon insult |
| CD11b (Mac-1) | **↑** | Peak levels 6-18hrs post trauma [27], | Peak levels 24hrs post insult |
| CD11b (Mac-1) | **↘** | Decreasing levels after 72 hours [28] | Decrease and restoration of expression at 72 hours after trauma |
| CD62L (L-selectin) | **↔ / (↗)** | Relatively unaltered or non-significant rise after trauma [33] or burns [25] | Non-significant rise upon insult |
| CD16 (FcyRIII) | n/a. | Decreased, during first 48 hours after polytrauma [34] | Gradual increase of membrane expression |
| CD16 (FcyRIII) | **↗** | Increase, after 48 hours post-trauma [48] | Gradual increase of membrane expression |
| CD32 (FcyRII) | **↔** | Unaltered after trauma [42] | Unaltered after insult |

Legenda: ↗, receptor expression increases upon insult; ↘, receptor expression decreases upon insult; ↑, increased receptor expression value measured after insult insult; ↔, receptor expression was unaltered upon insult. n/a., not applicable: no integral effect/different effect in human and pig studies.
